# A micro-patterned silicon chip as sample holder for macromolecular crystallography experiments with minimal background scattering

**DOI:** 10.1038/srep10451

**Published:** 2015-05-29

**Authors:** P. Roedig, I. Vartiainen, R. Duman, S. Panneerselvam, N. Stübe, O. Lorbeer, M. Warmer, G. Sutton, D. I. Stuart, E. Weckert, C. David, A. Wagner, A. Meents

**Affiliations:** 1 Deutsches Elektronen-Synchrotron DESY, Photon Science, Notkestraße 85, 22607 Hamburg; 2 Paul Scherrer Institut, Villigen PSI, 5232, Switzerland; 3 Diamond Light Source Ltd., Diamond House, Harwell Science & Innovation Campus, Didcot, Oxfordshire, OX11 0DE, United Kingdom; 4 Division of Structural Biology, Wellcome Trust Centre for Human Genetics, University of Oxford, Oxford, OX3 7BN, United Kingdom

## Abstract

At low emittance synchrotron sources it has become possible to perform structure determinations from the measurement of multiple microcrystals which were previously considered too small for diffraction experiments. Conventional mounting techniques do not fulfill the requirements of these new experiments. They significantly contribute to background scattering and it is difficult to locate the crystals, making them incompatible with automated serial crystallography. We have developed a micro-fabricated sample holder from single crystalline silicon with micropores, which carries up to thousands of crystals and significantly reduces the background scattering level. For loading, the suspended microcrystals are pipetted onto the chip and excess mother liquor is subsequently soaked off through the micropores. Crystals larger than the pore size are retained and arrange themselves according to the micropore pattern. Using our chip we were able to collect 1.5 Å high resolution diffraction data from protein microcrystals with sizes of 4 micrometers and smaller.

Structure determinations from microcrystals at low emittance synchrotron sources^1^,^2^,^3^,^4^ and X-ray free electron lasers (XFELs)^5^,^6^,^7^ have recently attracted a great deal of attention and have the potential to change the way X-ray crystallographic data is collected from macromolecular crystals in the future. In such experiments a large number of photons is focused into very small spots of a few square micrometers, enabling structure determination using measurements from multiple crystals in a reasonable time^2^,^3^,^8^. Recently phasing experiments from multi-crystal measurements have also been successfully performed^9^. At these new sources, the crystal lifetime is strongly limited by radiation damage and is typically in the range of a few seconds at synchrotron sources and restricted to single shots in the case of XFELs. These short measurement times not only allow for extremely fast data collections, but also offer new opportunities for structure determinations from microcrystals and for kinetic studies^10^. A common bottleneck for all these new experiments at both low emittance synchrotron sources and XFELs are fast and efficient sample delivery techniques.

In traditional X-ray crystallography, with crystals of sizes between a few tens to several hundreds of micrometers, individual crystals are typically mounted in nylon loops^11^. This mounting technique requires a considerable amount of time for sample preparation. Individual crystals are harvested out of their mother liquor and subsequently flash frozen by plunging them into liquid nitrogen to prevent them from dehydration and to reduce radiation damage effects. Since each loop ideally carries only one or a few crystals, automatic sample exchange using a robotic sample changer has become standard at most synchrotron beamlines. A more modern approach is the use of so called ‘micromounts’ and ‘micromeshes’ made from polyimide, with which the current practice is usually to work with several tens to hundreds of crystals on a single sample mount^8^,^12^.

A different sample delivery approach has been developed for serial femtosecond crystallography (SFX) experiments at XFELs. With present repetition rates of up to 120 Hz, further increasing to 4.5 MHz within a burst at the future European XFEL^13^, the sample delivery should ideally be synchronized to the XFEL pulses. Currently, a suspension of microcrystals is delivered to the X-ray beam using a continuous liquid jet. Using aerodynamic focusing, stable jet sizes with diameters down to a few micrometers have been realized^14^. Due to the small diameter of the liquid jets, larger crystals – which would provide a much stronger diffraction signal – need to be filtered out prior to the measurement in order to prevent clogging of the jet nozzle. Another drawback of continuous liquid jets for current SFX experiments is the large sample consumption, as the hit rates achieved so far are only on the order of 10%^7^. Presently, tens to hundreds of milligrams of macromolecular crystals are typically required for a structure determination^15^. Thus many attempts to reduce the required sample amount have been tried. One successful approach is to deliver the sample in a ‘tooth paste’-like lipidic cubic phase (LCP) jet^16^. In contrast to previous experiments, a successful structure determination using an LCP jet required only about 300 μg of protein directly crystallized in LCP^15^. The use of LCP jets for SFX is in particular advantageous when only small sample amounts are available.

Another method for fast sample delivery at both synchrotrons and XFELs involves the use of fixed substrates, which are mounted on mechanical translation stages and raster-scanned through the beam^17^,^18^. A promising approach with samples on fixed substrates is the use of micro-patterned chips where the crystals arrange themselves in a periodic array of wells^18^. The chip developed by Zarrine-Afsar *et al.* consists of a silicon mesh sealed with a polyimide film on both sides in order to prevent the crystals from dehydration. It is designed for room temperature measurements in a defined atmosphere. Depending on the dimensions the chip can harvest several hundreds of crystals. The patterned structure of the chip facilitates automatic measurements by raster scanning of the individual wells of the chip.

## Results

### Chip design

**Figure 1:**
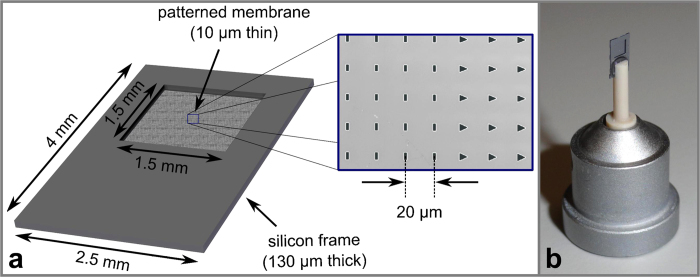
Schematic of the overall chip design with dimensions (**a**) and electron micrograph showing a few of the micropores of different shapes (magnified section in the center) and chip mounted on magnetic caps to be used for cryogenic data collection at existing macromolecular crystallography beamlines (**b**).

Following this approach, we have developed a microchip from single crystalline silicon with a periodic structure of micropores that serves as sample holder for several thousand microcrystals. The design of the micro-patterned chip is shown in Fig. 1a. It is entirely made from single crystalline silicon and consists of a frame with dimensions of 4 × 2.5 mm^2^ and a thickness of 130 μm and an inner membrane part of 1.5 × 1.5 mm^2^ with a thickness of 10–30 μm, depending on the design. The membrane part features a few hundred to several thousands of micropores. For the experiments described here, chips with micropore diameters of 2–5 μm with a pitch of 20 μm in both directions were chosen. In general the size and pitch of the pores can be matched to the crystal and X-ray beam size, respectively. Pore sizes down to 1 μm, with a pitch of 3 μm, have already been successfully realized by us. In order to avoid a preferred orientation of the microcrystals, often resulting in incomplete X-ray intensity datasets, the micropores are of different size and geometry, as shown in Fig. 1a. The micropores are generated by electron beam lithography and subsequent reactive ion etching. A light microscopy image of the chip with its membrane part is shown in Supplementary Fig. S1 online. A detailed description of the chip production is provided in the Methods section.

The chip is designed to work at cryogenic temperatures. Working at low temperatures has the significant advantage that, compared to room temperature measurements, the solvent water is in a vitrified state and thus crystals are protected from drying out by evaporation of the solvent and also radiation damage effects are reduced^19^. The chip dimensions are chosen such that the chip is compatible with most conventional open-flow cryocoolers installed at macromolecular crystallography beamlines. With its dimensions perpendicular to the goniometer axis smaller than the diameter of the cryostream, it is assured that the chip, in all positions, is permanently and fully exposed to the cold stream. Thus all crystals are always kept at cryogenic temperatures. In order to further prevent conductive heating of the chip by its mount adapter, which is typically at room temperature, the chip is glued onto the tip of a thermally insulating plastic pin attached to a standard mount used in macromolecular crystallography. Using this approach and with chip dimensions smaller than the inner cold gas stream of the cryojet we did not observe any ice formation on the chip nor on the base. The chip design further allows easy mounting onto the goniometer since conventional cryotools such as vials and cryotongues can be used. The chip mounted on a standard magnetic cap is shown in Fig. 1b. It can be mounted at any crystallography beamline and is compatible with most sample changing robots.

### Sample loading

**Figure 2:**
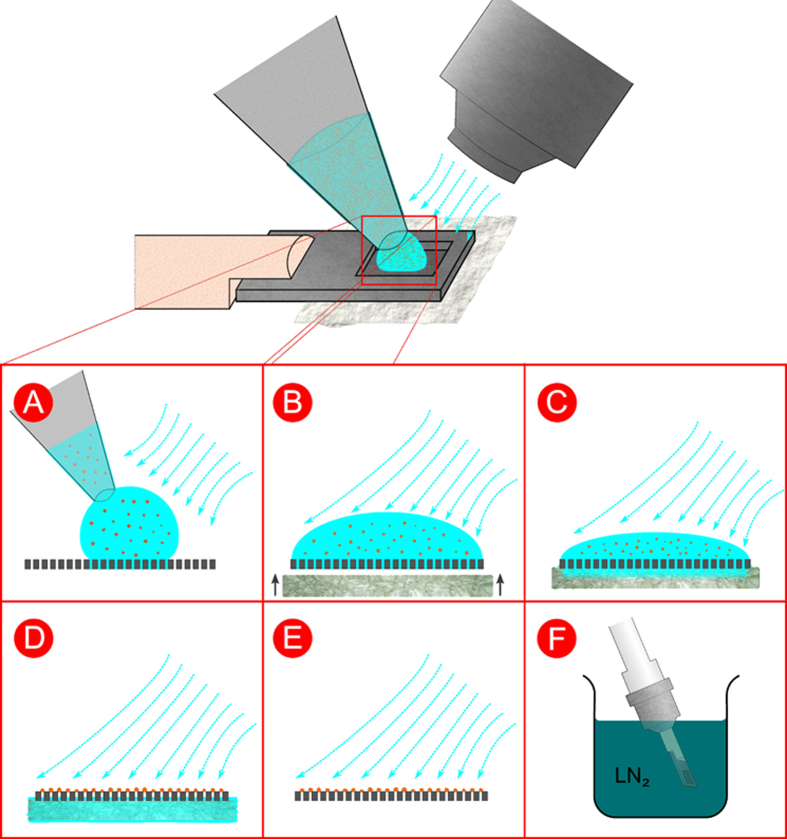
Sample loading procedure: A droplet of about 3 μl crystal suspension with a concentration of 1000–2000 crystals/μl is pipetted on the front-side of the chip (**A**). Due to capillary action the mother liquor fills the microchannels of the chip and forms a meniscus on the lower side (**B**). The mother liquor of the crystal suspension is removed by touching the lower side with a small wedge of filter paper which soaks off the liquid through the microchannels (**C**). Crystals with sizes larger than the pores are retained and arrange themselves in a periodic way according to the pore structure on the chip (**D**). The wet filter paper is then removed (**E**). Finally the chip with the crystals is flash-frozen by plunging it into liquid nitrogen (**F**). During this handling procedure the chip is placed in a continuous stream of air with a controlled degree of humidity in order to prevent drying of the crystals.

The procedure of loading microcrystals on the silicon chip is illustrated in Fig. 2. A suspension of the microcrystals, with a volume of typically 1–3 μl and a crystal density of 1000–2000 microcrystals per microliter, is pipetted onto the chip. Due to capillary action, the pores are immediately filled with mother liquor. By attaching a wedge of filter paper from the lower side of the chip, the mother liquor is soaked off through the micropores. Microcrystals larger than the pore diameter are retained and stay on the upper side of the chip. During sample loading, the chip is constantly exposed to an air stream with controlled humidity to prevent the crystals from drying out and thereby losing their diffraction properties^20^. In case of more viscous microcrystal suspensions, sample loading and liquid removal can be supported by suction as described in the Methods section and shown in Supplementary Fig. S2 online. Directly after crystal loading, the chip is flash-frozen by plunging it into liquid nitrogen. Due to the good thermal conductivity of silicon and the fact that most of the mother liquor is removed in an efficient way, we expect that crystals smaller than 20 μm do not require any cryoprotection and can be directly flash-frozen as reported for thin biological samples in cryo-electron microscopy^21^ and recently also shown for X-ray crystallography^22^. High occupation densities of the pores with crystals can be achieved using this procedure. The resulting periodic arrangement of the crystals in the pores allows for an automated raster scan approach for X-ray data collection.

### Background contribution from the chip

A critical parameter in X-ray micro-crystallography is the ratio of the diffraction signal to the corresponding background, which is even more important in cases of very small crystals and/or large-unit-cell systems, such as viruses or large molecular complexes. The intensity scattered into Bragg reflections (signal) is proportional to the illuminated crystal volume. The background is mainly determined by the amount of surrounding material such as mother liquor, polyimide supports, or nylon loops being exposed to the X-ray beam. Especially in case of smaller crystals on relatively thick supports, the Bragg intensity is buried in the background. This is particularly severe in structure determinations from microcrystals using LCP jets, which typically have a jet diameter of 20–50 μm. The scattering from the LCP phase significantly contributes to the background on the detector and drastically reduces the achievable signal-to-noise level of the Bragg reflections.

Compared to other materials the use of single crystalline silicon has the important advantage that the chip itself does not contribute to any background scattering signal. The background level is further reduced due to the efficient removal of the surrounding mother liquor, achievable with our chip.

**Figure 3:**
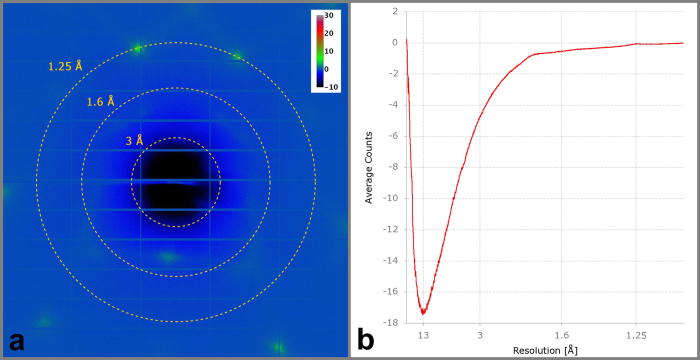
X-ray background level originating from the silicon chip. The image was obtained by taking the difference between images with and without chip in the full X-ray beam of beamline I02 (with a photon flux of 3 × 10^12^ ph/sec) and averaging over 50 difference images (**a**). The radial distribution of the azimuthally averaged difference signal is shown as a function of resolution (**b**). Negative values are caused by absorption effects of the silicon chip material.

We have measured the background contribution due to the chip by taking a series of fifty (50) images with and without the chip in the full X-ray beam of beamline I02 at Diamond Light Source, UK. Images were taken with a photon flux of 3 × 10^12^ ph/sec, a sample to detector distance of 156 mm, and an exposure time of 0.1 sec per image with a rotation increment of 0.01 degree at a photon energy of 12 keV. For each angular position the difference signal between the images with and without the chip was calculated and averaged over the whole series (shown in Fig. 3a). At certain positions regions with slightly higher intensity can be observed which can be attributed to thermal diffuse scattering from the single crystalline silicon. In our experiments scattering is caused by the (220), (331) and (333) Bragg reflections of silicon at resolutions of 1.92 Å, 1.25 Å and 1.05 Å, respectively. In Fig. 3b, we plot the azimuthally averaged intensity difference (I_with chip_ – I_without chip_) as a function of resolution, which corresponds to the radial distance on the detector with respect to the primary beam position. It can be seen that the difference signal is always below zero which indicates that for this exposure time the chip contributes no background photons. The difference signal is negative due to absorption by the silicon chip (which is less than 5% at energies larger than 12 keV). By using small rotation increments around a certain orientation of the chip for data collection it is possible to completely avoid the appearance of strong silicon Bragg reflections. Larger rotations of up to 60 degree can be realized but are accompanied by strong silicon Bragg reflections which potentially can cause damage to the detector. These normally appear at angles greater than typical Bragg reflections from macromolecular crystals (see Supplementary Fig. S3 online). In case of crystals diffracting to higher resolution the silicon reflections have to be excluded from data processing.

### X-ray structure determinations from microcrystals

**Figure 4:**
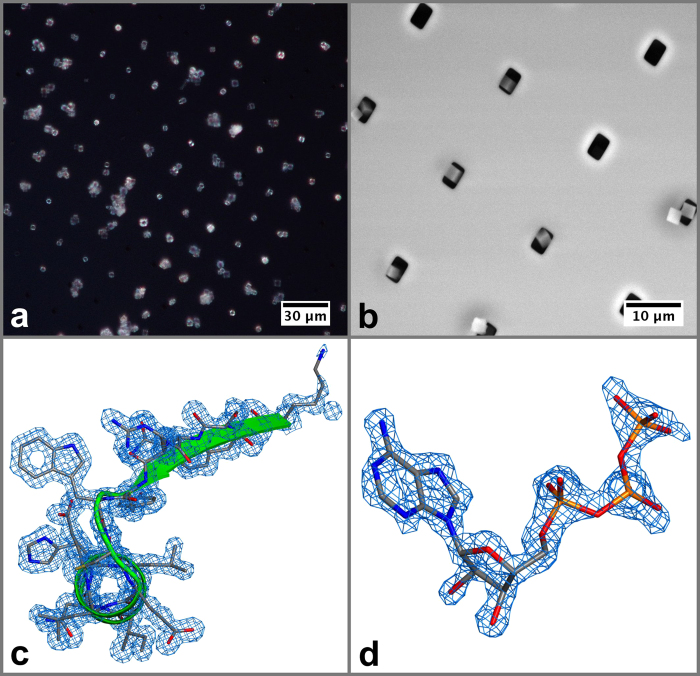
Images of CPV18 microcrystals with a size of up to 4 μm loaded onto the silicon chip as observed with differential interference contrast microscopy (**a**) and environmental scanning electron microscopy (**b**). The resulting high quality electron density (2F_o_ - F_c_ contoured at 1σ) obtained by X-ray micro-diffraction provides a high level of details as shown for a portion of the CPV polypeptide chain (**c**) and a bound ATP (**d**).

To prove the suitability of our chip for protein micro-crystallography, polyhedrin and lysozyme microcrystals of less than 4 μm in size were chosen as test samples for X-ray diffraction experiments. A light microscopy image and an electron micrograph of polyhedrin crystals loaded onto our chip, demonstrating the achievable high occupation densities of the pores, are shown in Fig. 4a and 4b, respectively. X-ray data collections from both protein crystals were performed at the microfocus beamline I24 at Diamond Light Source at a temperature of 100 K with a photon flux of ~2 × 10^11^ ph/sec and an X-ray beam size of a 7 × 7 μm^2^ (FWHM) at a photon energy of 12.8 keV. Locations of microcrystals were identified by raster scanning the chip through the X-ray beam and analysis of the corresponding diffraction signals as described in Ref. 23. Further data collection details are provided in Table 1 and in the Methods section. In both cases data integration was performed using XDS, data scaling and merging were performed with XSCALE^24^.

The polyhedrin microcrystals diffracted to a high resolution of 1.5 Å. A total number of 51 datasets were collected at different positions on the chip of which 23 datasets could be indexed and 22 were merged into the final dataset. Integration was performed using the first 4 degrees of crystal rotation corresponding to a dose of 28.5 MGy per crystal as calculated with RADDOSE-3D^25^. The structure of *Operophtera brumata* CPV18 polyhedrin was solved by molecular replacement with Phaser^26^, using the 3D coordinates of the *Bombyx mori* cypovirus 1 polyhedrin structure (PDB 2OH5) as a model^1^, and refined with Phenix^27^. Further details are provided in Table 1. Examples of the resulting electron density are shown in Fig. 4c, 4d and provide a high level of structural detail. No obvious signs of radiation damage can be observed upon inspection of the F_o_-F_c_ difference electron densities.

The resulting OpbrCPV18 structure is very similar to the recently published structure of isolated OpbrCPV18 crystals (PDB 4OTS)^8^, with a calculated rms displacement of 0.04 Å for all 248 Cα atoms. The difference in cell parameters between the two structures is negligible (102.81 Å for our structure, as opposed to 102.79 Å for 4OTS). The high resolution limit of 1.5 Å achieved with seleno-methionine labeled microcrystals mounted on our silicon chip is significantly higher than the 1.7 Å obtained for 4OTS using conventional micromeshes as crystal mounts. Seleno-methionine labeled polyhedrin crystals are typically of lower quality and exhibit worse diffraction properties than their native counterparts. Thus, with similar data collection parameters, the higher resolution achieved in our experiment is very likely a consequence of the efficient removal of mother liquor and the use of single crystalline silicon as a low-background substrate for the chip.

**Figure 5:**
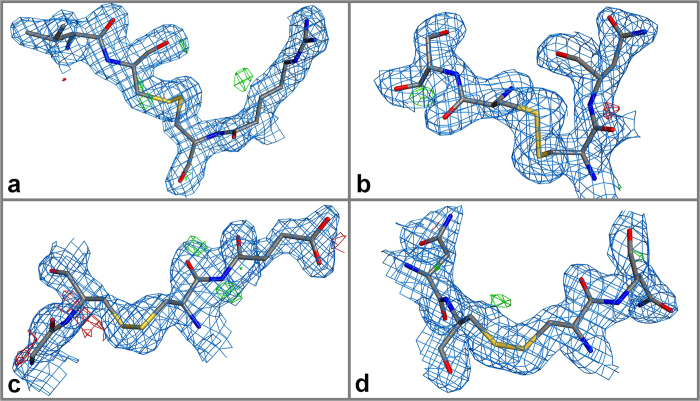
Composite (2F_o_ - F_c_, blue, contoured at 1σ) and difference (F_o_ - F_c_, green/red, contoured at 2.5σ) electron density maps of the four disulphide bridges of the refined lysozyme structure. Panels show the disulphide bridges between residues Cys 30 and Cys 115 (**a**), Cys 64 and Cys 80 (**b**), Cys 6 and Cys 127 (**c**), and Cys 76 and Cys 94 (**d**). Electron difference maps show no indication of specific radiation damage.

The hen egg white lysozyme microcrystals diffracted to a resolution of 2.1 Å. In total 139 datasets were collected on different positions of the chip from which 110 could be indexed. The first 3 degrees of every dataset were used for data integration, corresponding to a dose of 20.2 MGy per crystal. Due to non-isomorphism of the individual lysozyme microcrystals hierarchical clustering procedures were applied to find the best combination of datasets from isomorphous crystals^9^,^28^. Using this approach 73 datasets were merged into the final dataset (see Methods section and Supplementary Fig. S4 online for details). The structure was solved by molecular replacement using the lysozyme model PDB 193L^29^ as a template with Phaser^26^, manually built with Coot^30^, and refined with Phenix^27^. Further details can be found in Table 1 and a representative electron density map from lysozyme is provided in Supplementary Fig. S5 online. Electron density difference maps of the four disulphide bridges, as shown in Fig. 5, display no signs of specific radiation damage.

## Discussion

We have developed a new sample holder for macromolecular crystallography with a broad applicability ranging from very small to medium sized crystals. The chip can be loaded in a simple and efficient way. An entire crystallization drop can be pipetted onto the chip and the mother liquor is removed by blotting and capillary action of the micropores. All solid material larger than the pore diameter is retained by the chip and can be subjected to X-ray investigation. In this way no potential crystalline material is wasted, as is the case with conventional sample holders where it is very difficult to harvest all crystals from a crystallization drop into a loop.

The use of single-crystalline silicon and the efficient removal of the mother liquor lead to exceptionally low background scattering caused by the chip. As a result diffraction data collected from seleno-methionine labeled polyhedrin microcrystals loaded onto our chip are of superior quality compared to diffraction data from conventionally mounted native crystals.

The doses applied to the crystals, 28.5 MGy in case of polyhedrin and 20.2 MGy in case of lysozyme, are below the acceptable dose limits for cryocrystallography as reported in the literature^31^. Consequently no obvious signs of radiation damage could be observed. The structure determinations are based on measurements of 52 polyhedrin and 139 lysozyme microcrystals with sizes of 4 micrometer and smaller. Compared to room temperature serial crystallography experiments at both synchrotrons and XFELs the number of crystals required for a structure determination using our chip - at cryogenic temperatures - is significantly smaller. This offers new opportunities for structure determinations from microcrystals in particular if only a limited number of crystals is available or in case of very radiation sensitive crystals by measuring a larger number of crystals at a reduced dose. An often reported drawback of cryogenic data collection is the need for finding suitable cryoconditions. Using our chip this drawback does not hold since the microcrystals can be directly flash cooled on the chip without the need of any cryoprotectants.

The high thermal conductivity of single crystalline silicon further makes this chip ideally suited for future in-vacuum diffraction experiments, which are currently planned at several synchrotron sources. As open-flow cryostats cannot be used in vacuum, sample holders with good thermal conduction properties are required.

We have successfully shown the application of our single crystalline silicon chip for synchrotron experiments. In addition, the application of the chip has a large potential for experiments at XFELs, especially in cases where only a limited amount of sample is available or for experiments which require minimal background. The latter is especially important for nanometer-sized or 2D-crystals.

The application of the chip is not limited to macromolecular crystallography. It also can be applied to organic and inorganic crystals and may additionally be used for imaging and X-ray spectroscopy applications. In all cases, sample loading can be performed in the same simple and efficient manner as described for macromolecular crystals.

## Methods

### Chip Fabrication

The chips were fabricated from standard double side polished silicon <100> wafers with a thickness of 130 μm. The windows for the inner membrane part with a size of 1.5 × 1.5 mm^2^ were defined onto a photoresist mask by photolithography. The windows were then etched to a thickness of –10 μm by reactive ion etching (RIE) using the Bosch process. After removal of the residual photoresist mask, the flat side of the wafer was coated with a thin chromium layer using physical vapor deposition and spin coated with a Poly(methyl-methacrylate) (PMMA) resist. The pattern for the micropores was subsequently defined by electron beam lithography. The holes into the chromium hard mask were etched using RIE with Cl_2_/O_2_ chemistry. Finally the pores were etched through the silicon membrane by RIE using a SF_6_/C_4_F_8_ chemistry. The remaining chromium layer was removed by dry etching. A light microscopy image of the membrane part of chip with the micropores is shown in Supplementary Fig. S1 online.

### Sample preparation

Based on the Gene Bank sequence DQ192250^32^ the polyhedrin gene for Operophtera brumata CPV18 (OpbuCPV18) was synthesized by GeneArt (Life Technologies). The polyhedrin gene was amplified, inserted into the transfer vector pBacPAK9 (Clontech) and recombinant baculovirus was produced by co-transfection of linearised baculovirus DNA and the transfer vector following a standard procedure^33^. Expression and purification of polyhedra followed the method described by Anduleit^34^. Briefly cells were harvested 96 hours post-infection and lysed by dounce homogenisation in 5 mM Hepes, pH 7.5. Cell components and debris were removed by centrifugation for 5 mins at 1,000 × g. Polyhedra crystals were enriched by successive washing and centrifugation. Seleno-methionine-labeled polyhedra were generated as published previously^4^ and the incorporation of Seleno-mehionin was confirmed to be around 80% using mass spectroscopy. Polyhedrin microcrystals were suspended in a 50% ethylene-glycol solution and further diluted before application to the chip.

Hen egg white lysozyme microcrystals were grown by batch crystallization from a 50 mg/ml lysozyme solution in 0.1 M NaAc buffer (pH 4.0) and 8% NaCl as precipitant. The microcrystal suspension was diluted before application to the chip. No cryoprotectants were added before flash-cooling.

### Chip Loading

A photograph of the setup for sample loading of the chip is shown in Supplementary Fig. S2 online. The silicon chip itself is glued onto a plastic pin which is attached to a standard magnetic cap (as shown in Fig. 1b). The magnetic cap carrying the empty chip is attached to a magnet on translation stages which allow well defined positioning of the chip in the controlled humidity stream of the HC1 device^20^. Once the correct humidity is adjusted 1–3 μl of crystal suspension is pipetted onto the chip. By attaching a wedge of filter paper at the lower side of the chip (as described in Fig. 2) the mother liquor is soaked off through the micropores. Depending on the viscosity of the solution this process can take up to several seconds.

For suspensions with higher viscosity this process can be further supported by air suction. For this purpose the chip is slightly lowered in vertical position and placed on a porous material such as poly-ethylene or polypropylene (shown in Supplementary Fig. S2 online). A vacuum pump is attached to the lower side of the porous material which sucks the liquid through the micropores of the chip. The time which is required to remove the mother liquor through the pores can be adjusted by changing the low-pressure. Using this approach even very viscous liquids can be fully removed from the chip. It has to be mentioned here that the suction has to be switched off quickly after removal of the mother liquor because crystals tend to dry out faster in the air draft and thereby lose their diffracting properties.

After removal of the mother liquor the chip with the crystals is immediately flash-frozen by plunging it into liquid nitrogen. The chip can be then protected with a vial for further handling and transportation.

### Data Collection

Diffraction data sets from both lysozyme and polyhedrin crystals were taken at the microfocus beamline I24 at Diamond Light Source. The diffraction images were obtained with a beamsize of 7 × 7 μm^2^ (h x v) at a photon energy of 12.8 keV. Measurements were performed at 100 K using an open flow cryostat. The silicon chip carrying the crystals was mounted whilst maintaining cryogenic temperatures. Alignment of the chip in the X-ray beam was performed using the on-axis light microscope available at beamline I24.

### Data Processing

For the polyhedrin crystals a total number of 51 datasets, each comprising a 5° sweep of 100 images with an oscillation range of 0.05° per image, were collected at different positions on the chip; 23 datasets could be indexed and 22 of these were finally merged. Data integration was performed with XDS, using the first 4 degrees of every sweep. Scaling and merging of datasets was done with XSCALE^24^. All correlation coefficients between datasets were larger than 94%, indicating a high degree of isomorphism. The structure solution was obtained by molecular replacement, with Phaser^26^, using the 3D coordinates of the Bombyx mori cypovirus 1 polyhedrin structure as a model (PDB 2OH5), and refined with phenix.refine^27^. Stereo views of residues 120–150 of the resulting electron density maps are provided in Supplementary Fig. S5–S9 online.

For the lysozyme crystals 139 datasets were recorded from the individual microcrystals. For each dataset 120 diffraction images were taken while rotating the silicon chip by 6° in total (which corresponds to an oscillation range of 0.05° per image). The first 60 images of each single dataset were used for further data analysis, performed with the XDS package^24^. Using XDS 110 datasets, out of the 139 measured, could be indexed. Each dataset contained about 620 indexed reflections on average. For each dataset an individual resolution limit was determined according to the conventional criterium I/σ(I) > 2 for the highest resolution shell and only reflections up to this resolution limit were included in the further analysis. A large variance of the mutual correlation coefficients between the datasets was observed which is a strong indication for non-isomorphism of the lysozyme microcrystals. In order to find the best possible combination of datasets from isomorphous crystals we applied a hierarchical clustering analysis, as suggested in recent publications dealing with multi-crystal data^15^,^28^. For that we used the corresponding functions available in the SciPy clustering package for Python. The clustering procedure was performed based on the intensity correlation between the datasets. We therefore defined *d*_*ij*_ = 1 – *c*_*ij*_ as the ‘distance’ between two datasets, where *c*_*ij*_ is the intensity correlation coefficient between the common Bragg reflections of dataset *i* and *j*. The number of common reflections between two datasets thereby varied between 0 and several hundred and was about 60 on average. The correlation coefficients vary between 1 (intensities fully correlated) and –1 (fully uncorrelated) and were calculated using the program XSCALE^24^, which was also used for the merging of the multiple datasets. Hereby datasets with a high degree of correlation were combined to several clusters until a total number of 11 clusters were obtained (this number is mainly owed to the computing power which was available to the project). During clustering newly formed clusters were linked pursuant to the ‘average’ method, also known as the Unweighted Pair Group Method with Arithmetic Mean (UPGMA). The hierarchical clustering procedure is illustrated in a so-called dendrogram, shown in Supplementary Fig. S4 online. 11 clusters were formed according to their corphenetic distances as described in the documentation of the SciPy function ‘fcluster’ (criterion ‘maxclust’).

Out of the 11 formed clusters all possible combinations, which are 2^11^ = 2048 in total, were merged and refined in order to find the best final selection. The statistics of the resulting dataset are provided in Supplementary Table S10 online. Since the structure for lysozyme is known, molecular replacement and automatic refinement could be performed for each selection, with the PHENIX software suite^26^,^27^ using as template the structure with PDBID 193L^29^. The *R*_*free*_ value was taken as the main criterium in order to judge the quality of the particular combination of datasets. However, data completeness and also R values, like *R*_*meas*_,were taken as further criteria. Finally, a selection of 57 datasets, with minimum values of *R*_*work*_/*R*_*free*_ = 0.188/0.231 was chosen for the final refinement. Structure refinement was performed up to a resolution of 2.1 Å. The resulting electron density map is shown in Supplementary Fig. S5 online.

## Additional Information

**How to cite this article**: Roedig, P. *et al.* A micro-patterned silicon chip as sample holder for macromolecular crystallography experiments with minimal background scattering. *Sci. Rep.* **5**, 10451; doi: 10.1038/srep10451 (2015).

**Accession Codes**: Solved structures were deposited in the Protein Data Bank (PDB) under PDB IDs 4×35 and 4×3B for CPV18 and lysozyme, respectively.

## Supplementary Material

Supplementary Information

## Figures and Tables

**Table 1:** Data collection and refinement statistics (molecular replacement).

	**Polyhedrin (CPV18)**	**Lysozyme**
**Data collection**		
Space group	*I*23 (197)	*P*4_3_2_1_2 (96)
Cell dimensions		
*a*, *b*, *c* (Å)	102.81, 102.81, 102.81	78.38, 78.35, 37.79
α, β, γ (°)	90, 90, 90	90, 90, 90
Resolution (Å)	80 – 1.5 (1.6 – 1.5)^1^	19.55 – 2.1 (2.21 – 2.1)^1^
*R*_merge_	19.6 (47.9)	23.9 (40.6)
*CC*_*1/2*_	0.988 (0.775)	0.973(0.773)
*I*/σ*(I)*	7.7 (2.4)	5.76 (2.29)
Completeness (%)	96.8 (89)	94.8 (89.4)
Redundancy	7.7 (3.8)	7.7 (3.8)
		
**Refinement**		
Resolution (Å)	36.35 – 1.5	19.55 – 2.1
No. reflections	28126	6777
*R*_work_/*R*_free_	0.126/0.169	0.186/0.229
No. atoms		
Protein	2039	1009
Nucleotide	63	-
Ion	3	7
Water	216	105
*B*-factors^2^		
Protein	6.26	26.10
Nucleotide	26.36 (GTP)	-
	15.35 (ATP)	-
Ion	27.36	38.82
Water	15.10	29.40
R.m.s. deviations		
Bond lengths (Å)	0.006	0.006
Bond angles (°)	1.159	0.92

^1^Values in parentheses are for highest-resolution shell.

^2^B_eq_ as atomic displacement parameters for CPV18 were refined anisotropically.
