# Supplementary material for: A micro-patterned silicon chip as sample holder for macromolecular crystallography experiments with minimal background scattering
Source: Sci Rep. 2015 May 29;5:10451. doi: 10.1038/srep10451 (PMC4448500; doi:10.1038/srep10451)
Supplement: Supplementary Information [file srep10451-s1.pdf]

A micro-patterned silicon chip as sample holder for macromolecular crystallography experiments with minimal background scattering

Supplementary Information

P. Roedig[1], I. Vartiainen[2], R. Duman[3], S. Panneerselvam[1], N. Stuebe[1], O. Lorbeer[1], M. Warmer[1], G. Sutton[4], D. I. Stuart[3,4], E. Weckert[1], C. David[2], A. Wagner[3], and A. Meents[1*]

[1]Deutsches Elektronen Synchrotron DESY, Photon Science, Notkestraße 86, 22607 Hamburg
[2]Paul Scherrer Institut, Villigen PSI, 5323, Switzerland
[3]Diamond Light Source Ltd., Diamond House, Harwell Science & Innovation Campus, Didcot, Oxfordshire, OX11 0DE, United Kingdom
[4]Division of Structural Biology, Wellcome Trust Centre for Human Genetics, University of Oxford, Oxford, OX3 7BN, United Kingdom

*Correspondence: alke.meents@desy.de

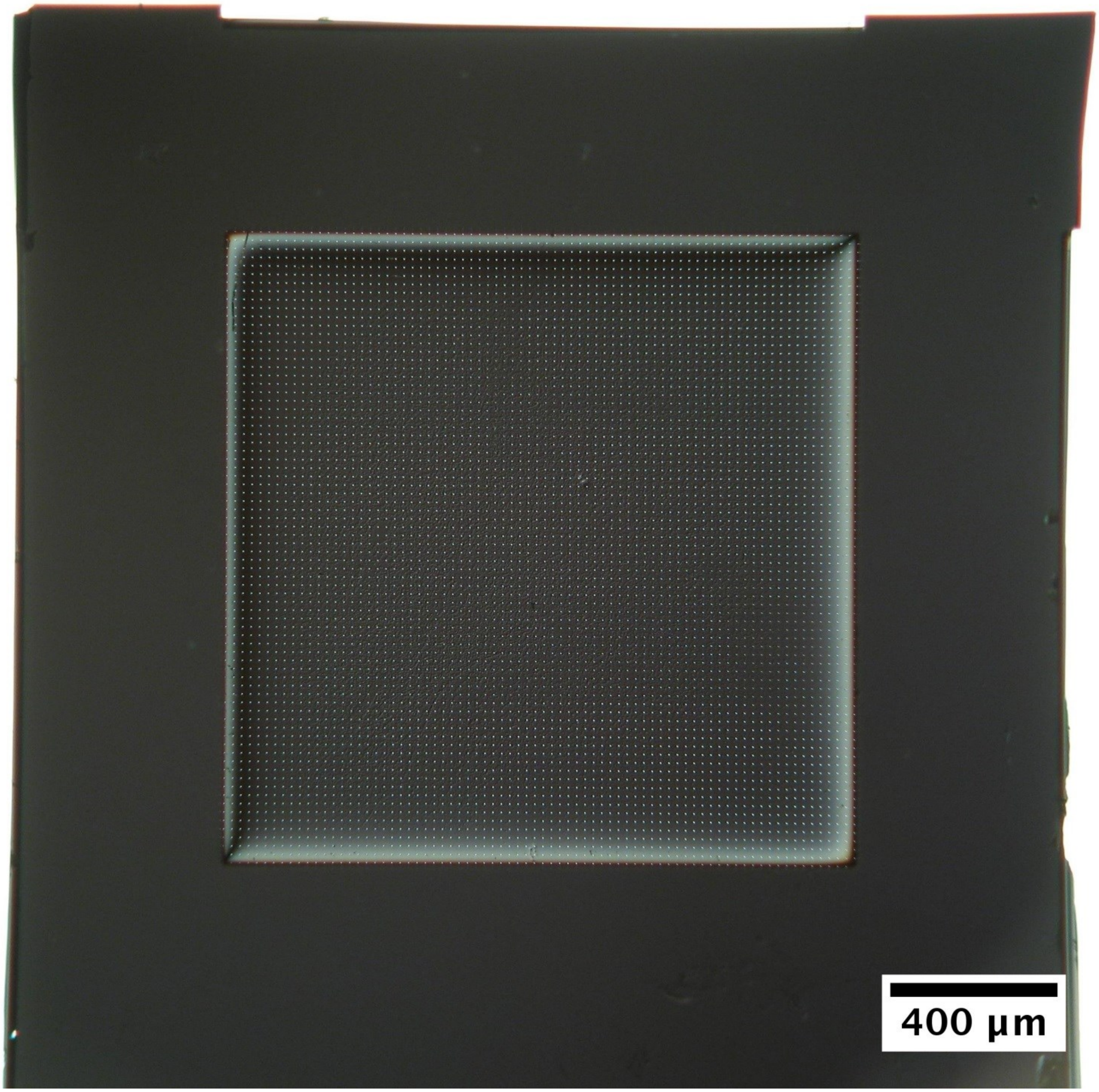

Supplementary Figure S1: Light microscope image of the upper part of the silicon chip holder. The chip is designed as an outer frame of single silicon with dimensions of 4 x 2.5 mm^2 and a thickness of 100 µm and an inner silicon membrane part of 1.5 x 1.5 mm^2 with a thickness of 10 – 30 µm, depending on the design. In the above figure the membrane part consists of more than 5000 small micropores with a diameter of 3 µm and a period of 20 µm.

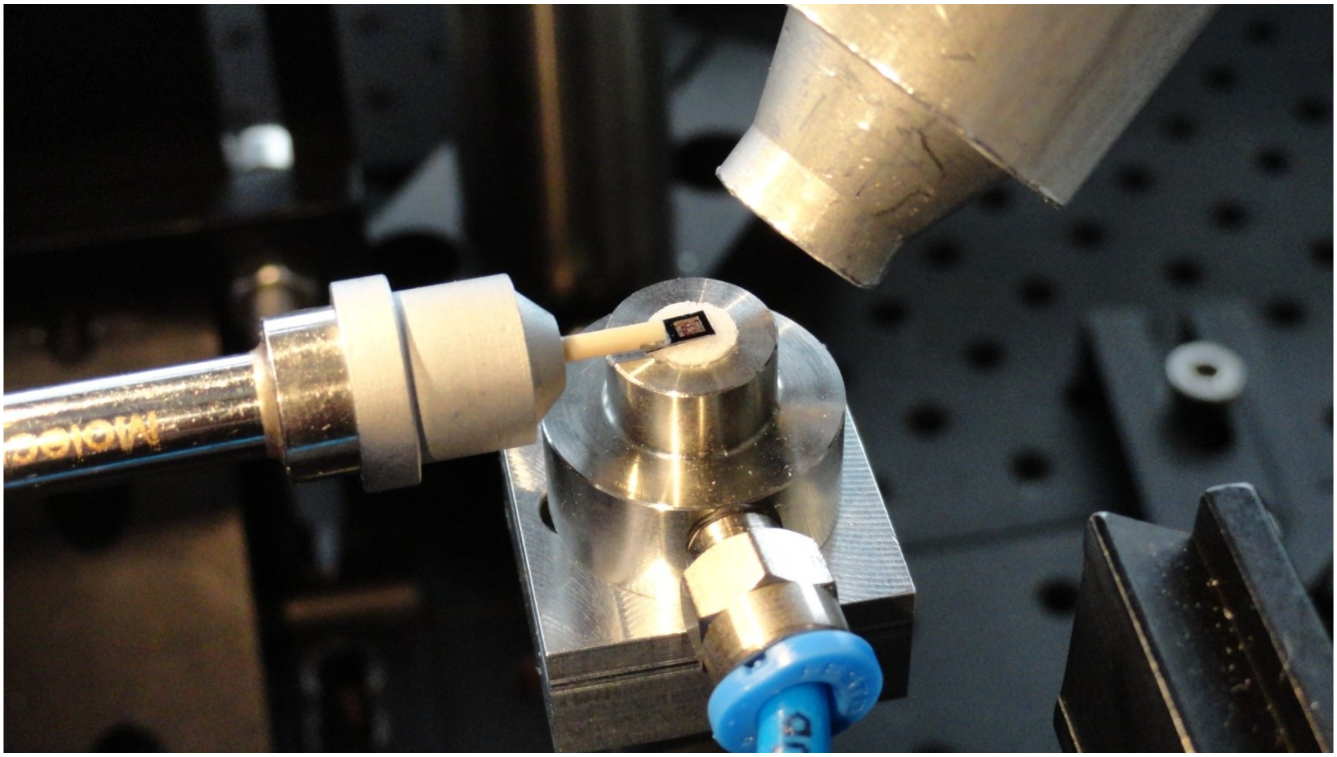

Supplementary Figure S2: Setup for chip loading. The chip is fixed to a standard magnetic cap and positioned in the humidity stream (coming from the top right) to prevent dehydration of the crystals. A drop of 1 – 3 µl of crystal suspension is pipetted onto the chip and the mother liquor is either removed by attaching a filter paper from underneath (not shown) or by air suction (shown).

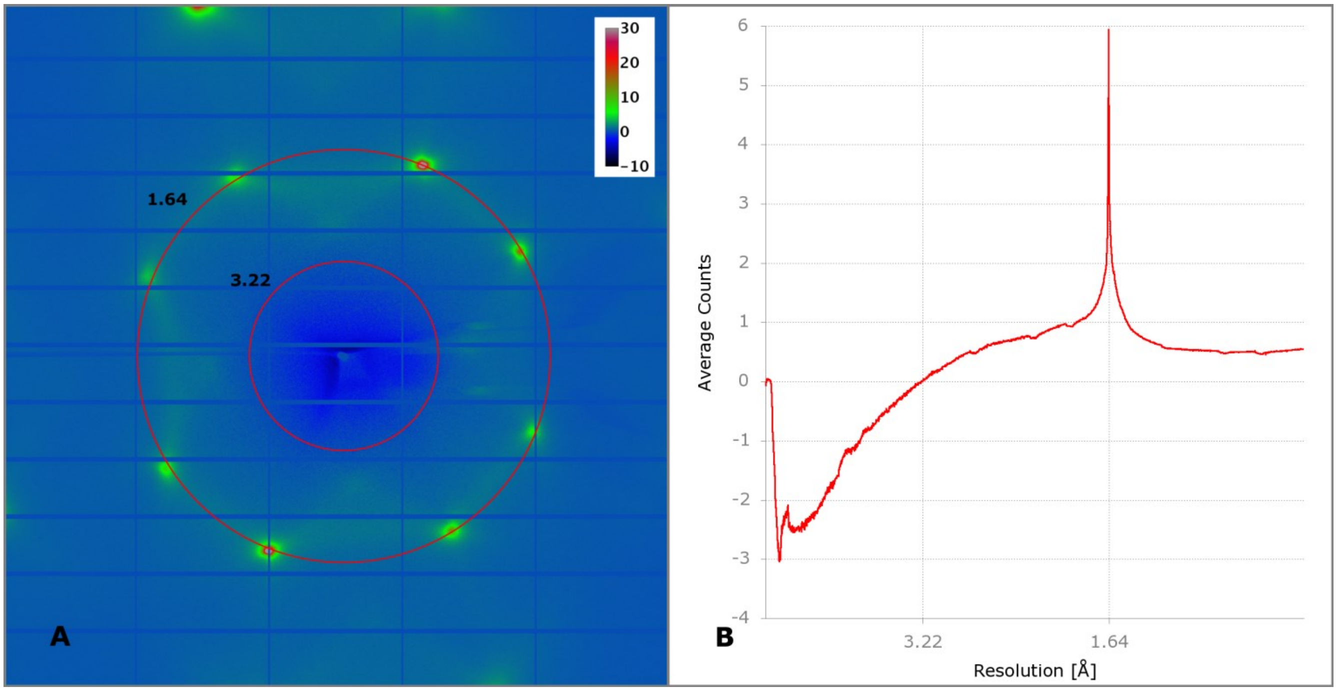

Supplementary Figure S3: Background signal at beamline I24 at an energy of 12.398 keV, a photon flux of 3.5 x 10^{11} ph/sec and an exposure time of 500 msec (A). The image was obtained by taking the difference between images with and without chip in the X-ray beam and averaging over 120 images (6° rotation in total). The radial distribution of the azimuthally averaged difference signal is shown as a function of resolution (B).

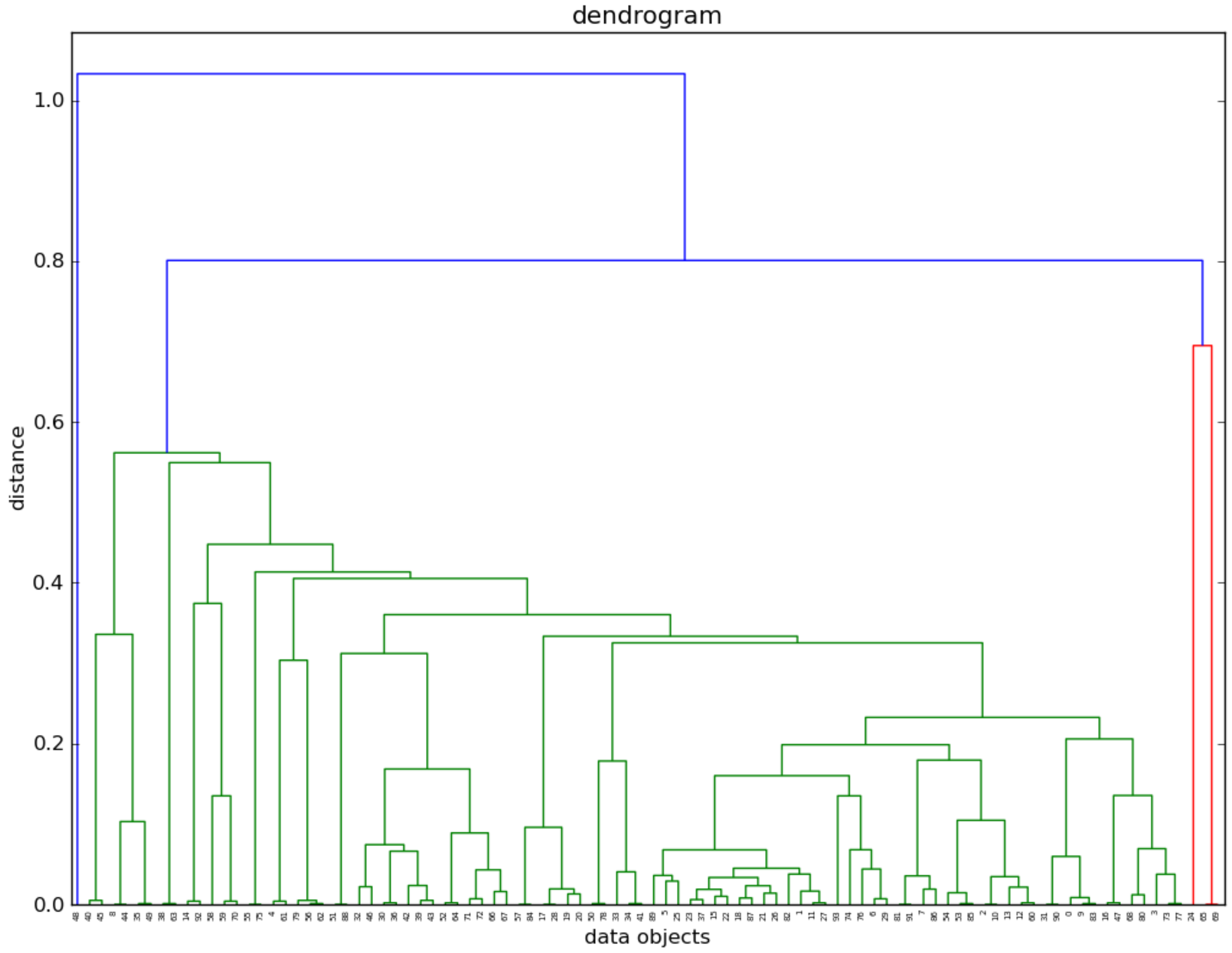

Supplementary Figure S4: Dendrogram for the hierarchical clustering of the lysozyme datasets. The clustering was performed according to the intensity correlation of common Bragg reflections. The distance between two datasets was defined as $d_{ij} = 1 - c_{ij}$, where c_{ij} is the corresponding intensity correlation coefficient.

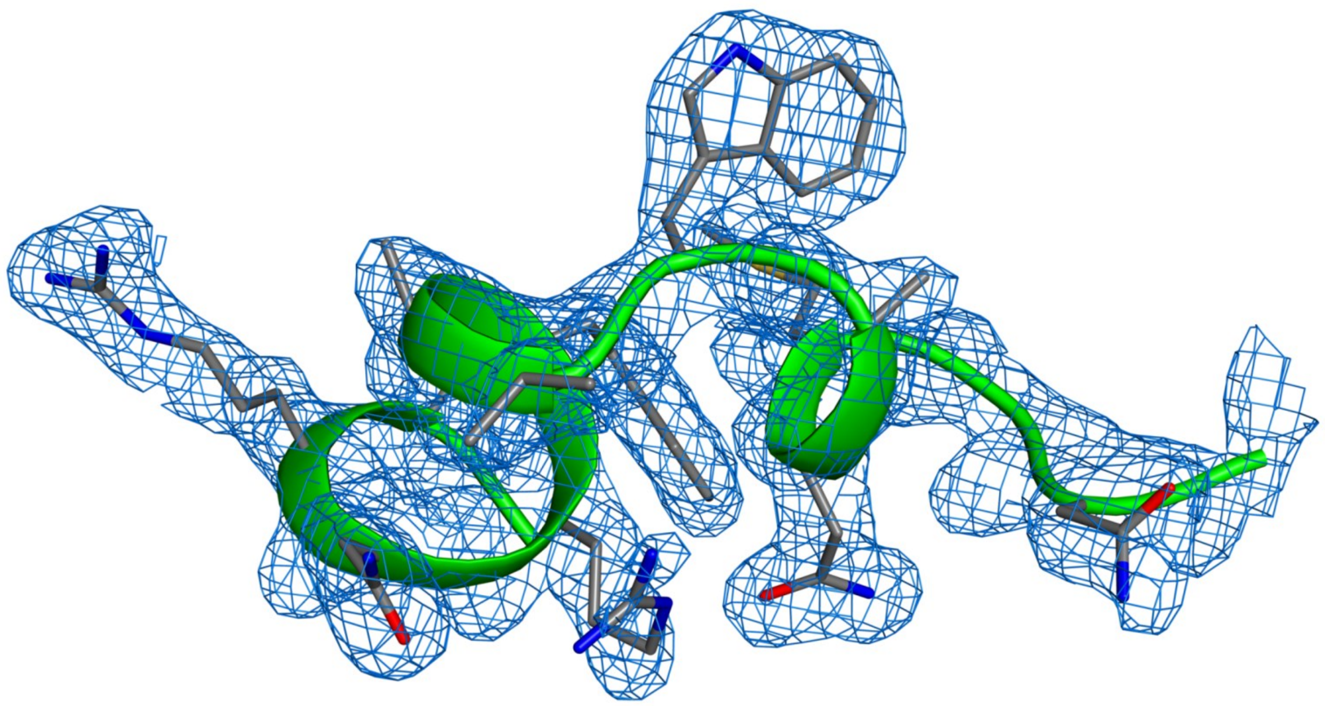

Supplementary Figure S5: Electron density map of the refined lysozyme structure (2Fo-Fc, refinement up to 2.1 Å resolution, contoured at sigma level 1). The main chain is illustrated in cartoon representation and the side chains are shown as sticks.

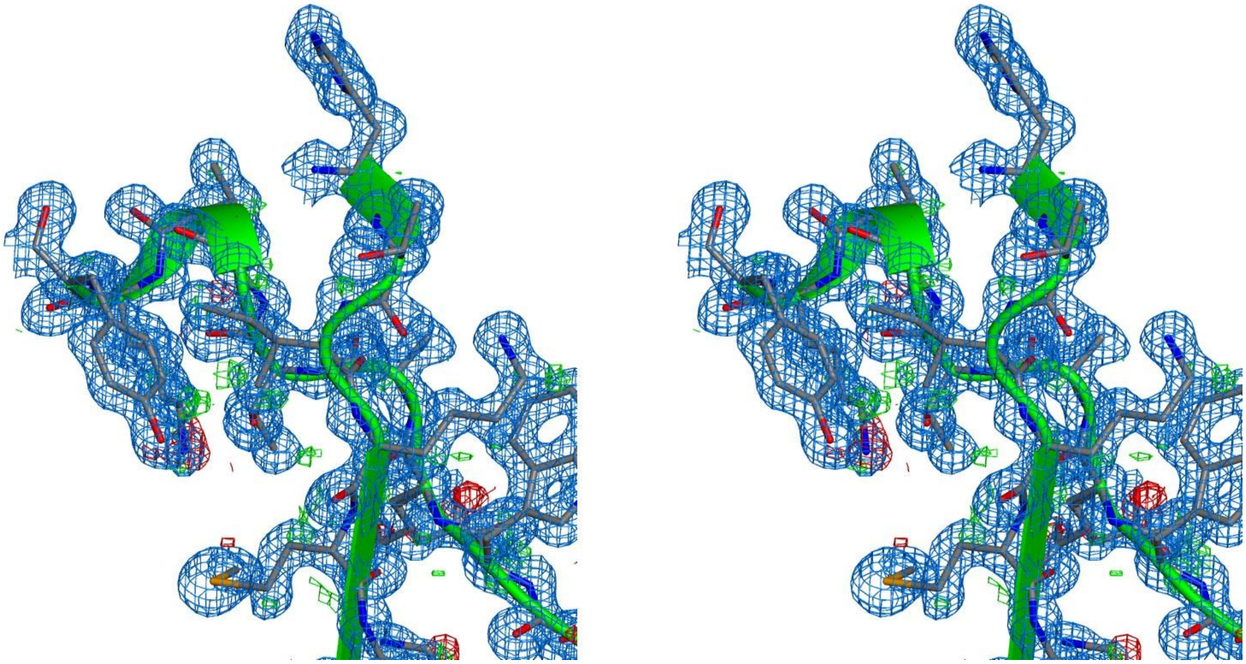

Supplementary Figure S6: Stereo view (orientation 1) of a representative region of the electron density of OpbrCPV18, showing the 2F0-Fc density (sigma level 1) in blue and Fo-Fc density (sigma level 2.5) in red and green. The corresponding alpha carbon backbone, for amino acids 120-150, is depicted in green.

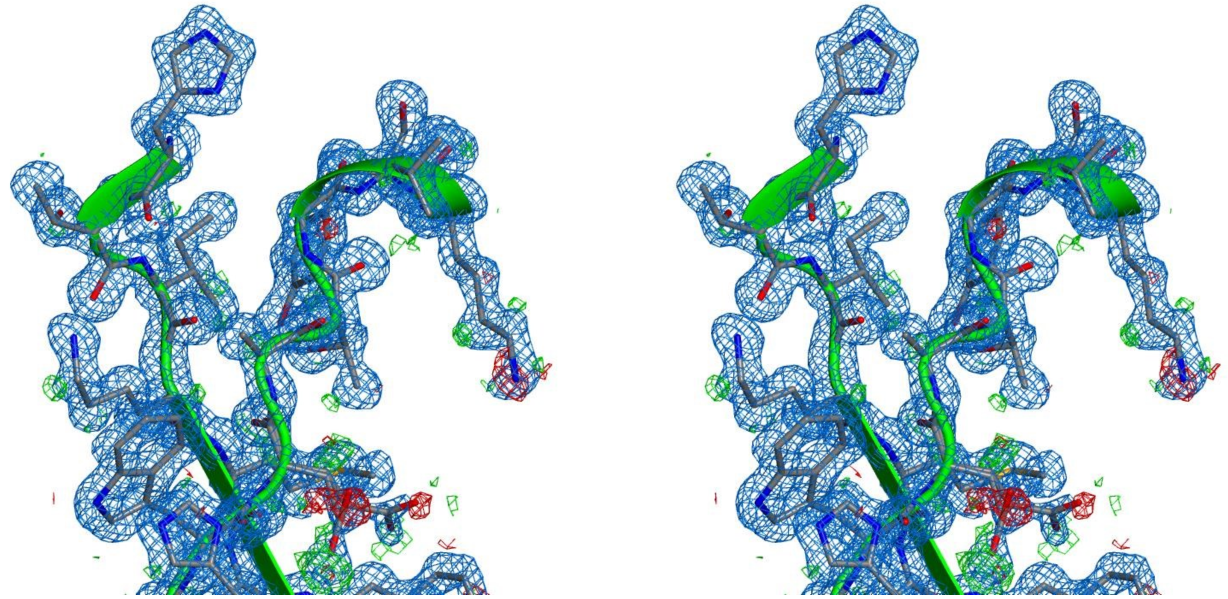

Supplementary Figure S7: Stereo view (orientation 2) of a representative region of the electron density of OpbrCPV18, showing the 2F0-Fc density (sigma level 1) in blue and Fo-Fc density (sigma level 2.5) in red and green. The corresponding alpha carbon backbone, for amino acids 120-150, is depicted in green.

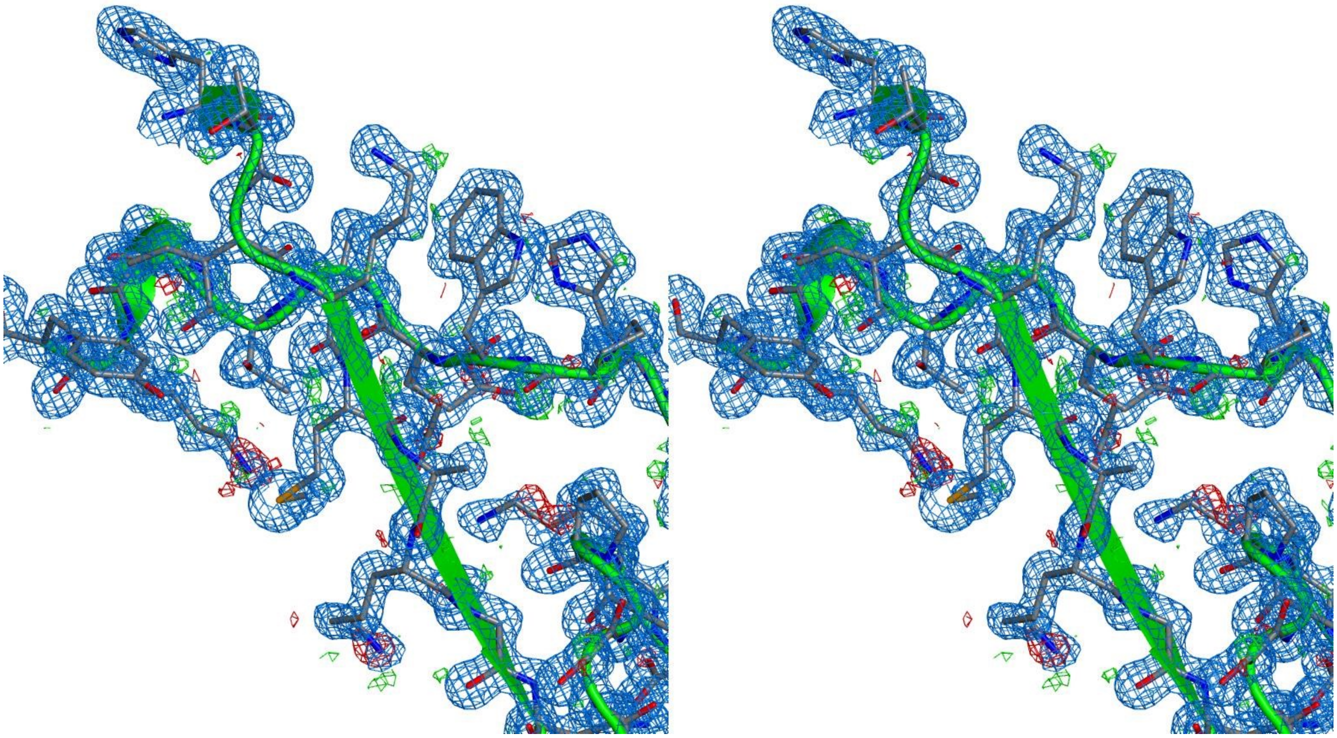

Supplementary Figure S8: Stereo view (orientation 3) of a representative region of the electron density of OpbrCPV18, showing the 2F0-Fc density (sigma level 1) in blue and Fo-Fc density (sigma level 2.5) in red and green. The corresponding alpha carbon backbone, for amino acids 120-150, is depicted in green.

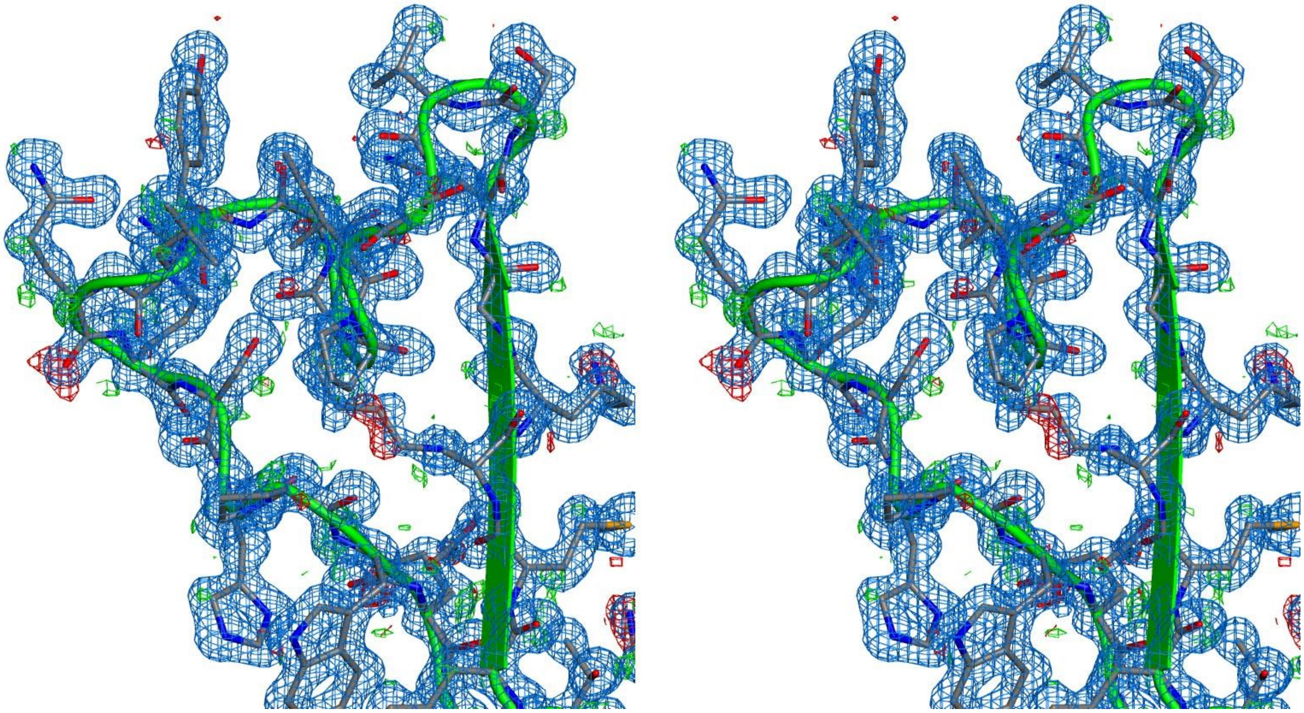

Supplementary Figure S9: Stereo view (orientation 4) of a representative region of the electron density of OpbrCPV18, showing the 2F0-Fc density (sigma level 1) in blue and Fo-Fc density (sigma level 2.5) in red and green. The corresponding alpha carbon backbone, for amino acids 120-150, is depicted in green.

Supplementary Table S10: Data statistics of the final lysozyme dataset (73 datasets merged) for the various resolution shells.

subset of resolution limit [Å]	number of reflections			completeness of data	I/σ(I)	R_{meas}	$CC_{1/2}$
	observed	unique	possible				
15	159	16	30	53.3 %	14.20	14.6 %	98.8
10	607	58	58	100.0 %	13.97	14.2 %	98.9
6	3078	268	272	98.5 %	11.57	16.8 %	98.4
4	9464	761	768	99.1 %	10.89	22.9 %	97.7
3.5	6221	517	518	99.8 %	9.82	31.2 %	95.3
3	10079	908	916	99.1 %	7.81	29.8 %	95.6
2.8	4052	559	565	98.9 %	5.35	31.5 %	95.0
2.5	7196	1151	1193	96.5 %	4.37	34.7 %	90.5
2.3	5422	1051	1186	88.6 %	3.55	38.3 %	88.6
2.2	2944	674	760	88.7 %	3.04	46.5 %	66.3
2.1	3031	814	910	89.5 %	2.51	46.3 %	75.1
**total**	**52253**	**6777**	**7176**	**94.4 %**	**5.97**	**26.3 %**	**97.7**
